# MEGADOCK-on-Colab: an easy-to-use protein–protein docking tool on Google Colaboratory

**DOI:** 10.1186/s13104-023-06505-w

**Published:** 2023-09-22

**Authors:** Masahito Ohue

**Affiliations:** Department of Computer Science, School of Computing, Tokyo Institute of Technology, 4259-G3-56 Nagatsutacho, Midori-ku, Yokohama, Kanagawa 226-8501 Japan

**Keywords:** Protein–protein docking, Protein–protein interaction, MEGADOCK, Google Colaboratory

## Abstract

**Motivation:**

Since the advent of ColabFold, numerous software packages have been provided with Google Colaboratory-compatible ipynb files, allowing users to effortlessly test and reproduce results without the need for local installation or configuration. MEGADOCK, a protein–protein docking tool, is particularly well-suited for Google Colaboratory due to its lightweight computations and GPU acceleration capabilities. To increase accessibility and promote widespread use, it is crucial to provide a computing environment compatible with Google Colaboratory.

**Results:**

In this study, we report the development of a Google Colaboratory environment for running our protein–protein docking software, MEGADOCK. We provide a comprehensive ipynb file, including the compilation of MEGADOCK with the FFTW library installation on Colaboratory, the introduction of related tools using PyPI/apt, and the execution and visualization of docking structures. This streamlined environment enables users to visualize docking structures with just one click. The code is available under a CC-BY NC 4.0 license from https://github.com/ohuelab/MEGADOCK-on-Colab.

## Introduction

Understanding protein–protein interactions is of paramount importance for elucidating complex biological phenomena and identifying potential drug targets. MEGADOCK [1, 2] is a protein docking software developed by our team to accurately predict protein–protein interactions. Designed to function on Linux-based systems, MEGADOCK supports GPU computing via CUDA and parallel computing on cluster machines. These capabilities render it particularly suitable for conducting comprehensive prediction validations, including one-to-many and many-to-many protein interactions [3]. In this paper, we introduce MEGADOCK-on-Colab, a tool that facilitates the execution of MEGADOCK on Google Colaboratory (hereinafter referred to as Colaboratory).

Colaboratory (https://colab.research.google.com) is a Jupyter Notebook hosting service provided by Google Research, enabling users to compose and execute Python code directly within their web browsers. This versatile service caters to a wide range of applications, including machine learning, data analysis, and education, while offering complimentary access to accelerators such as GPUs. Each computing environment is provisioned as a virtual machine (VM) with root privileges and is subsequently discarded after use, thereby permitting flexible installation of libraries and external tools. The implementation of AlphaFold2 [4] and ColabFold [5] via Colaboratory has enticed a growing number of life science researchers to utilize the platform. Notably, since the emergence of ColabFold, an increasing array of software packages have been accompanied by Colaboratory-compatible.ipynb files, enabling users to effortlessly test and reproduce results without necessitating local installation or configuration (e.g., small-molecule docking software DiffDock [6]).

In this study, we have successfully established a Colaboratory environment for executing MEGADOCK. We furnish a comprehensive ipynb file encompassing the compilation of MEGADOCK, inclusive of the installation of the FFTW library on Colaboratory, the incorporation of pertinent tools via PyPI/apt, and the execution and visualization of docking structures within the Colaboratory framework. This streamlined environment empowers users to visualize docking structures with a single click.

## Implementation

The following is a step-by-step explanation of the MEGADOCK-on-Colab process flow and implementation method.

### Preparation for input

In Colaboratory, user interfaces can be added to Python variables using annotations such as #@param{type:”string”}. For instance, by writing:

R_pdb_id = ”1CGI” #@param {type:”string”}

An input form for the variable R_pdb_id can be displayed with the pre-filled value of “1CGI”. Using this approach, we have configured the tool to accept two PDB IDs and chain names as input forms. Additionally, we incorporated input forms to receive the main arguments for MEGADOCK. The reason for preparing these inputs prior to tool installation is to ensure that the input forms are displayed as close to the top of the Jupyter Notebook as possible, making users aware of their presence.

### Installation of MEGADOCK on Colaboratory VM

Next, we install the GPU version of MEGADOCK on the Colaboratory VM as follows. System commands can be used in Colaboratory by adding a ‘!’ prefix.

!git clone https://github.com/akiyamalab/MEGADOCK

!git clone https://github.com/NVIDIA/cuda-samples

!apt install -y libfftw3-dev libfftw3-single3

%cd ./MEGADOCK

!make -j 2 -f Makefile.colab

Since the operating system is based on Ubuntu, apt can be used (to determine the actual OS being used, run commands like !cat /etc/os-release). Note that the NVIDIA drivers and CUDA are pre-installed (to check the available GPU and CUDA version, execute !nvidia-smi).

### Downloading PDB files

We download the specified PDB files from the Protein Data Bank using wget and use Biopython [7] to extract the specified chains. Biopython is available on PyPI and can be installed as follows:

!pip install biopython

### Executing MEGADOCK

MEGADOCK can be executed with the following command:

!./megadock-gpu -R $MDPDBR -L $MDPDBL -t $MDt -N $MDN -o $MDOF

Please note that the arguments need to be passed from Python to the shell environment variables. For instance, we can set the values beforehand using something like:

os.environ[’MDPDBR’] = pdbr.

### Visualizing predictions

NGLView [8] is a molecular viewer that operates within Jupyter Notebook. It is available through PyPI and can be installed with the following command:

!pip install nglview

To visualize the output.pdb file, use the following code:

from google.colab import output

output.enable_custom_widget_manager()

import nglview as nv

view = nv.show_structure_file(”output.pdb”)

view

## Results

**Fig. 1:**
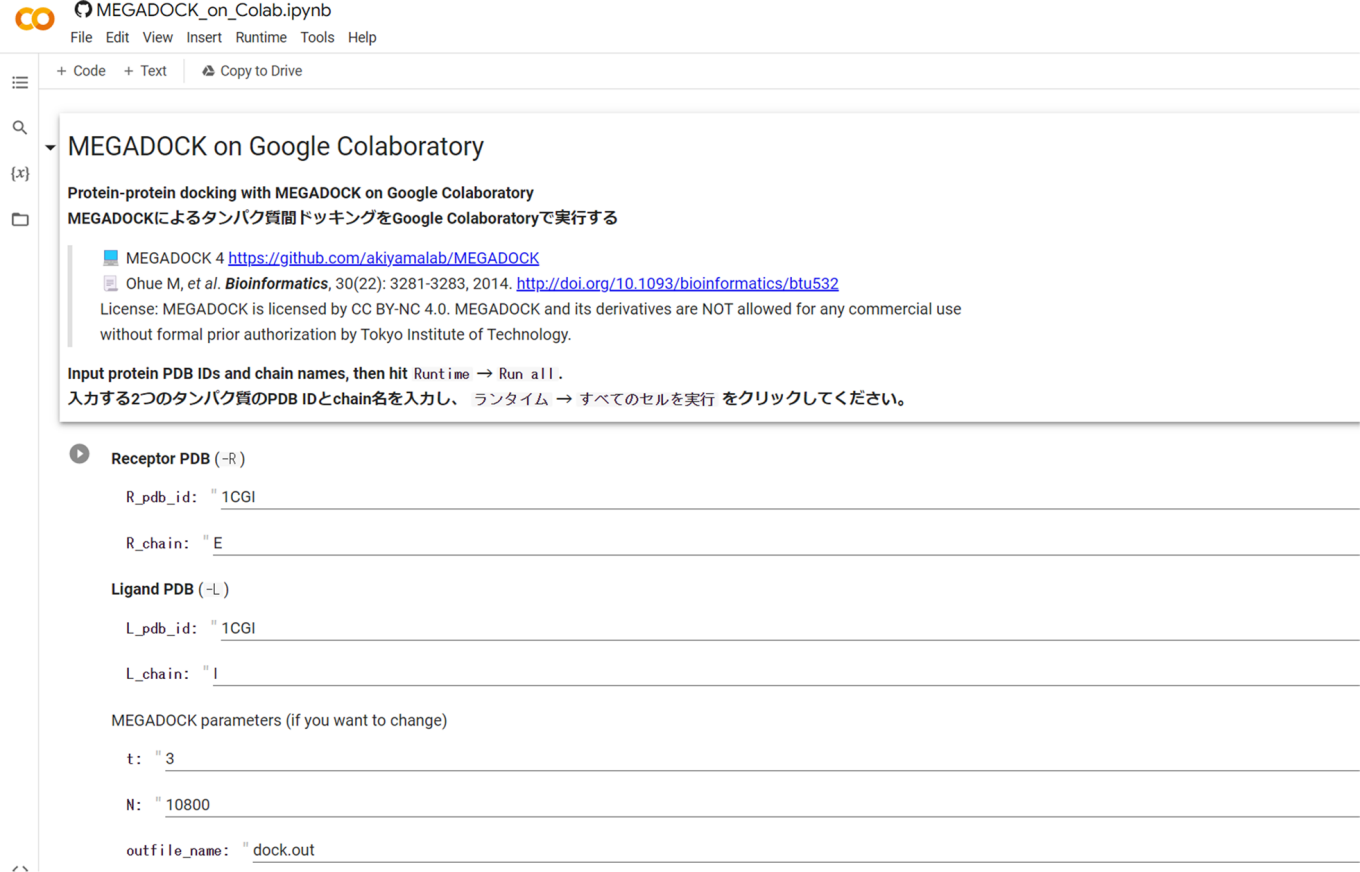
MEGADOCK-on-Colab input form

**Fig. 2:**
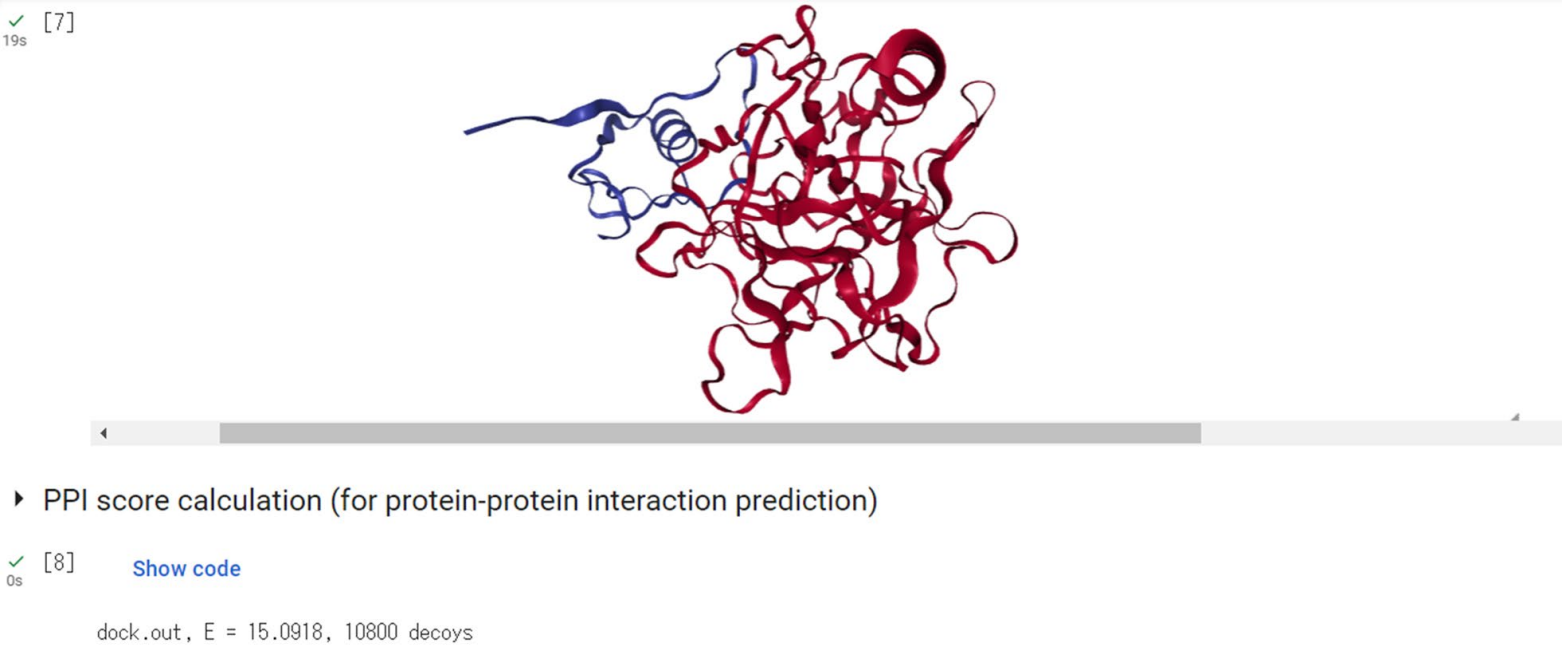
Visualization with NGLView in MEGADOCK-on-Colab

Figures 1 and 2 showcase example screenshots of MEGADOCK-on-Colab. Upon entering the requisite information, such as files and parameters, into the initial input form, users can automatically execute the entire series of processes by selecting the “Run all” option. As of April 2023, the free version of Colaboratory frequently assigns an NVIDIA Tesla T4 GPU to its virtual environments. Utilizing the Tesla T4 GPU, the MEGADOCK docking calculation for PDB 1CGI chain E (consisting of 245 residues) and 1CGI chain I (consisting of 56 residues) required approximately 5 s. The processing time for non-docking calculations, encompassing installation procedures, amounted to roughly 60 s. Additionally, although we have demonstrated how to execute the program using specified PDB IDs, it is also possible for users to upload their own PDB files and perform the docking calculations.

## Discussion

In this study, we have delineated a method for constructing an execution environment on Colaboratory, utilizing MEGADOCK as a representative example.

Owing to its virtual nature, Colaboratory alleviates concerns regarding potential damage to the environment arising from installation failures, while consistently offering a nearly identical computing environment. However, it’s important to note that Colaboratory does not guarantee complete reproducibility. The libraries, OS version, GPU, CPU, and other factors are determined by the cloud and can vary, potentially affecting the results of compiled code. Thus, while Colaboratory facilitates reproducibility to some extent, it does not ensure it in all circumstances, enhancing the convenience of software utilization.

As evidenced in the present study, Colaboratory supports not only Python-based programs but also the compilation of diverse programming languages, execution of binary files, and implementation of system commands, effectively operating as a GPU-equipped server. In light of the ongoing development of an extensive array of GPU-compatible libraries, including those for deep learning, sharing reproducible execution environments on Colaboratory is progressively becoming a standard practice in software publication.

## Limitations

While our study introduces significant advancements in protein–protein docking software, there are certain limitations that need to be acknowledged.

Firstly, the reproducibility aspect of Colaboratory is not absolute. Although Colaboratory offers a nearly identical computing environment, it does not guarantee complete reproducibility. Factors such as the libraries, OS version, GPU, and CPU are determined by the cloud and can vary, potentially affecting the results of compiled code. This inherent variability underscores the need for caution when interpreting results obtained solely from this platform.

Secondly, our current implementation does not delve deeply into the detailed analysis of protein–protein interaction surfaces. While we provide a general overview and visualization, intricate details, especially those crucial for understanding specific molecular interactions, are not extensively covered. This might limit the depth of analysis researchers can perform using our tool, especially when a nuanced understanding of interaction sites is required.

Lastly, the visualization capabilities of our tool, while robust, could benefit from the integration of more advanced molecular viewers. Tools like mol* [9] offer enhanced visualization features that can provide a more comprehensive and detailed view of molecular interactions. Incorporating such advanced viewers in future updates could significantly elevate the user experience and the depth of analysis possible.

In conclusion, while our tool offers a novel approach and several advantages, users should be aware of these limitations when utilizing it for their research. We aim to address these in future iterations, ensuring a more comprehensive and user-friendly experience.

## Data Availability

The code is available under a CC-BY NC 4.0 license from https://github.com/ohuelab/MEGADOCK-on-Colab.
